# Functional and Transcriptional Senescence Profiles of CD8^+^ T Cells Associate With the Presence of Carotid Plaques in People Living With HIV

**DOI:** 10.1093/infdis/jiag018

**Published:** 2026-01-07

**Authors:** Marc J T Blaauw, Adriana Navas, Victoria Rios Vásquez, Nadira Vadaq, Marvin A H Berrevoets, Twan Otten, Wilhelm A J W Vos, Louise E van Eekeren, Albert L Groenendijk, Gert Weijers, Esther Lutgens, Hans J P M Koenen, Marien I de Jonge, Mihai G Netea, Joost H W Rutten, Andre J A M van der Ven, Niels P Riksen

**Affiliations:** Department of Internal Medicine, Radboud University Medical Center, Nijmegen, The Netherlands; Department of Internal Medicine and Infectious Diseases, Elizabeth-Tweesteden Ziekenhuis, Tilburg, The Netherlands; Department of Internal Medicine, Radboud University Medical Center, Nijmegen, The Netherlands; Department of Internal Medicine, Radboud University Medical Center, Nijmegen, The Netherlands; Department of Internal Medicine, Radboud University Medical Center, Nijmegen, The Netherlands; Center for Tropical and Infectious Diseases (CENTRID), Faculty of Medicine, Diponegoro University, Dr. Kariadi Hospital, Semarang, Indonesia; Department of Internal Medicine and Infectious Diseases, Elizabeth-Tweesteden Ziekenhuis, Tilburg, The Netherlands; Department of Internal Medicine, Radboud University Medical Center, Nijmegen, The Netherlands; Department of Internal Medicine and Infectious Diseases, OLVG, Amsterdam, The Netherlands; Department of Internal Medicine, Radboud University Medical Center, Nijmegen, The Netherlands; Department of Internal Medicine and Department of Medical Microbiology and Infectious Diseases, ErasmusMC, Erasmus University, Rotterdam, The Netherlands; Medical UltraSound Imaging Center (MUSIC), division of Medical Imaging, Radboud University Medical Center, Nijmegen, The Netherlands; Department of Cardiovascular Medicine and Immunology, Mayo Clinic, Rochester, Minnesota, USA; Department of Laboratory Medicine: Laboratory for Medical Immunology, Radboud University Medical Center, Nijmegen, The Netherlands; Department of Laboratory Medicine: Laboratory for Medical Immunology, Radboud University Medical Center, Nijmegen, The Netherlands; Department of Internal Medicine, Radboud University Medical Center, Nijmegen, The Netherlands; Department of Immunology and Metabolism, Life and Medical Sciences Institute, University of Bonn, Bonn, Germany; Department of Internal Medicine, Radboud University Medical Center, Nijmegen, The Netherlands; Department of Internal Medicine, Radboud University Medical Center, Nijmegen, The Netherlands; Department of Internal Medicine, Radboud University Medical Center, Nijmegen, The Netherlands

**Keywords:** HIV-1, atherosclerosis, flow cytometry, cellular senescence, bulk transcriptomics

## Abstract

**Background:**

People living with HIV (PLHIV) are at increased risk of atherosclerotic cardiovascular disease (ASCVD), but the immunological mechanisms driving plaque formation remain unclear. This study investigated the association between peripheral immune cell subsets and carotid atherosclerotic plaques in PLHIV without prior clinical ASCVD.

**Methods:**

In this multi-center cross-sectional study, virally suppressed PLHIV receiving antiretroviral therapy were enrolled from two Dutch cohorts: a discovery cohort (n = 994) and a validation cohort (n = 200). Participants underwent carotid ultrasound imaging to assess plaques using standardized criteria. Immune profiling was performed using high-dimensional flow cytometry of 355 immune cell populations. Associations with plaque were analyzed using linear regression adjusted for relevant confounders, and key findings were validated in the second cohort. Transcriptomic profiles of main cell populations were evaluated using deconvoluted bulk RNA sequencing to identify differential expression and pathway enrichment.

**Results:**

Carotid plaques were present in 584 participants (49%)—502 of 994 (51%) in the discovery and 82 of 200 (41%) in the validation cohort. Plaques were associated with higher counts of several CD8^+^ T cell subsets, particularly PD-1-expressing cytotoxic T cells with an interferon secretion profile (CD8_TC1_PD1^+^). Transcriptomic analysis of CD8^+^ T cells revealed downregulated mitochondrial function and upregulated type 1 interferon and epidermal growth factor receptor signaling, indicating a senescent phenotype.

**Conclusions:**

CD8^+^ T cell senescence may contribute to early atherosclerotic plaque formation in PLHIV. Targeting immune senescence could offer a novel strategy for cardiovascular risk reduction in this population.

**Clinical Trials Registration:**

ClinicalTrials.gov Identifier: NCT03994835

## Teaser

In virally suppressed people with HIV, carotid plaques were linked higher PD-1^+^ cytotoxic CD8^+^ T cells with interferon activity and altered signaling, suggesting CD8^+^ T cell senescence may drive early atherosclerotic plaque development.

The introduction of combined antiretroviral therapy (cART) has significantly reduced mortality among people living with HIV-1 (PLHIV) [1]. However, with increased life expectancy, non-AIDS related conditions, such as atherosclerotic cardiovascular diseases (ASCVD), have become leading causes of death in this population [2]. In addition to traditional risk factors like smoking, dyslipidemia, and hypertension, chronic inflammation and immune activation are believed to contribute to the elevated cardiovascular risk in PLHIV [3]. Inflammatory markers such as soluble CD14 (sCD14), high-sensitive CRP, and interleukin-6 remain elevated despite viral suppression [4, 5]. Moreover, pro-inflammatory monocyte subsets persist even after successful cART [6]. This sustained inflammatory state may result from microbial translocation due to increased intestinal permeability [7] and coinfections with viruses such as cytomegalovirus (CMV), hepatitis B, and hepatitis C [8].

Carotid ultrasound is a noninvasive tool to detect atherosclerosis. Carotid plaques are established predictors of myocardial infarction in the general population [9], and PLHIV are at increased risk for their development [10]. In earlier work, we found that carotid plaques in PLHIV were associated with age and prior myocardial infarction but not with HIV-specific factors, such as CD4 nadir, duration of infection or treatment [11]. However, recent data suggest that residual viremia—detectable but unquantifiable plasma viral loads—is an independent risk factor for ASCVD (Otten T et al Nature Medicine revision under consideration, CROI 2024 K2-783). Individuals with both residual viremia and carotid plaques were more likely to develop ASCVD, highlighting a potential role for HIV-related immune dysregulation in plaque development and progression.

Atherosclerosis is a chronic inflammatory disease involving both innate and adaptive immunity, leading to the formation of immune cell-rich, lipid-laden plaques in large- and medium-sized arteries [12]. In this study, we hypothesized that HIV-induced alterations in circulating immune cells are associated with the increased cardiovascular risk in PLHIV. To examine this, we performed detailed immunophenotyping and functional characterization of circulating innate and adaptive immune cells in PLHIV with and without carotid plaques.

## METHODS

### Study Population and Design

The 2000HIV cohort is a Dutch multicenter observational cohort of PLHIV on suppressive cART, recruited between November 2019 and October 2021 from 4 specialized HIV clinics. It is part of the Human Functional Genomics project [13] with details described previously [14]. Inclusion criteria were HIV-1 positivity, age ≥18, on cART for ≥6 months and viral load <200 copies/mL. Exclusion criteria included pregnancy, acute or opportunistic infection, recent antibiotic use, active malignancy, or use of immunomodulatory drugs (n = 20).

The study was approved by the Medical Ethical Review committee region Arnhem-Nijmegen (NL68056.091.81) and registered on https://clinicaltrials.gov (ID: NTC03994835). Written informed consent was obtained from all participants.

### Carotid Ultrasound Measurements

Participants underwent carotid ultrasound (Mindray, DC80A, L14-5WE transducer) to assess plaque presence. A plaque was defined as a focal intima media thickness (IMT) >1.5 mm or >50% thicker that surrounding IMT in the carotid artery, per international consensus [15]. The IMT was measured in triplicate bilaterally, 1 cm distal to the bulb. All 4 ultrasound operators were uniformly trained and periodically reevaluated at Radboud UMC.

### Flow Cytometry Measurements

Fasting blood samples were collected and shipped overnight to Radboud UMC for centralized processing. Samples were immunophenotyped using 3 panels (17–20 markers) on a 21-color 6-laser CytoFLEX-LX (Beckman Coulter). Daily quality control and standardization was performed using CytoFLEX Daily QC Fluorospheres (Beckman Coulter, Catalog # B53230), CytoFLEX Daily IR QC Fluorospheres (Beckman Coulter, Catalog # C06147), and SPHEROtm Rainbow calibration particles 6-peak (Spherotech Inc., Catalog # RCP-30-5A-6). Data acquisition was performed using CytExpert software 2.3 (Beckman Coulter). Specific antibodies were selected for identification of 355 populations of the main innate, T and B cell subsets, and markers of activation, exhaustion, and intracellular interaction (eg, HLA-DR, PD1, CD38). A complete list of all antibodies used is displayed in Supplementary Tables 1–3. The optimization and standardization of the flow cytometry panels, together with the staining and manual gating strategy, have been described previously [16, 17]. Manual gating and analysis were performed in Kaluza V2.1.2 (Beckman Coulter). Antibodies and measured subsets are listed in Supplementary Figure 1.

### Bulk Transcriptomic Analysis and CD8^+^ T Cell Deconvolution

Bulk transcriptomic profiles were processed from peripheral blood mononuclear cells (PBMCs) of the full cohort. Comprehensive methodologies for data collection and sequencing technique are extensively detailed in prior publications [14, 18]. Quality control excluded 42 samples due to poor sequencing metrics, duplicate sequencing, sex mismatches, or incomplete metadata. The final dataset comprised 58 347 transcripts from 1857 high-quality samples (unique donors) available for deconvolution and downstream analysis. Based on results from association analyses of absolute cell counts and carotid plaque presence, we focused subsequent transcriptomic analysis on deconvoluted CD8^+^ T cells.

High-resolution CD8^+^ T cell deconvolution was performed using CIBERSORTx [19] on filtered RNA-seq data, excluding low-abundance transcripts. Gene annotations were applied using Ensembl BioMart [20], retaining biologically relevant genes. A custom CD8^+^ T cell reference signature matrix, derived from single-cell RNA-seq data from the same cohort [14, 21], enabled cell type-specific deconvolution. Cross-platform batch effects between the single-cell reference and bulk mixtures were mitigated using CIBERSORTx S-mode batch correction, with quantile normalization as recommended for RNA-seq data [19]. This approach generated a CD8^+^ T cell-specific expression matrix, which was used for downstream differential expression and pathway analyses.

### Statistical Analysis

The study included a discovery and validation cohort, enrolled at separate sites. Findings from the discovery cohort were subsequently tested for consistency in the validation cohort. Numerical data were reported as medians (interquartile range) and categorical variables as frequencies (%). Comparisons used *χ*^2^, Fisher's exact, or Wilcoxon rank-sum tests as appropriate.

Flow cytometry data underwent inverse rank transformation for normalization. A linear regression model, adjusted for potential confounders, was used to analyze associations between immune cell subsets (dependent variables) and carotid plaque presence (independent variable). The model was adjusted for age, sex, current smoking status, use of lipid-lowering medication, history of hypertension, metabolic syndrome, and Black ethnicity as clinically relevant confounders. In addition, principal component analysis of model residuals (Supplementary Figure 2) was used to identify additional potential confounders, based on adjusted *R*^2^ threshold of >0.03. Variables meeting this criterion were added in a multistep procedure and *a* ≥ 10% change in the β-coefficient of the exposure was used to define confounding. Seasonality emerged as a confounder and was included consistently in both cohorts. We found seasonality as a potential confounder for the association of immune cell subsets and presence of carotid plaques. This finding is supported by studies in which seasonality has a major impact on cytokine responses and inflammation in stimulated PBMCs of the Human Functional Genomics Project [22]. The seasonality correction was performed similar to Ter Horst et al [22].

Participants with prior cardiovascular events (eg, myocardial infarction, stroke) were excluded from plaque association analyses. Results were considered validated if they met a false discovery rate (FDR) adjusted *P* < .05 in the discovery cohort and nominal *P* < .05 in the validation cohort, with consistent direction of effect.

All statistical analyses were conducted using Rstudio v4.2.2.

### Differential Expression Analysis of CD8^+^ T Cell Transcriptomics

Differential expression analysis (DEA) was conducted to compare CD8^+^ T cell transcriptomes between participants with and without carotid plaques using DESeq2 workflow [23]. The model used negative binomial generalized linear models, followed by multiple testing correction via Independent Hypothesis Weighting and Log_2_FC shrinkage using the “apeglm” method. Confounders included age, sex, seasonality, ethnicity, and batch (plate).

Genes were considered differentially expressed if they met an FDR < 0.05 (discovery cohort) and nominal *P* < .05 (validation cohort) with consistent Log_2_FC direction.

### Pathway Enrichment Analysis

Gene set enrichment analysis (GSEA) was applied to ranked gene list from DEA, using a composite ranking score of −Log_10_(*P*) × sign (Log_2_FC). Multiple pathway databases were queried using the clusterProfiler package [24], including Hallmark, Kyoto Encyclopedia of Genes and Genomes, Gene Ontology (biological process [BP], molecular function, and cellular component [CC]), Reactome, WikiPathways, and Disease ontology.

Significant enrichment was defined using an adjusted *P* < .05 with Benjamini–Hochberg correction.

## RESULTS

### Baseline Characteristics

The study included a discovery cohort of 994 participants and a validation cohort of 200 participants. All had valid carotid ultrasound and immunophenotyping data and no prior cardiovascular events. More males were included in this study, 824 (83%) in the discovery cohort and 162 (81%) in the validation cohort. Carotid plaques were present in 502 of 994 (51%) in the discovery cohort and in 82 of 200 (41%) in the validation cohort, indicating a lower plaque prevalence in the validation cohort. In both cohorts, participants with carotid plaques were older (55 vs 45.5 years in the discovery cohort, and 56.5 vs 48.5 years in the validation cohort), more frequently using lipid-lowering therapy (16% vs 8% and 32% vs 8%) and had higher rates of former smoking (33% vs 25% and 44% vs 25%). They had lived long with HIV (14.6 vs 11.5 years and 13.7 vs 10.4 years) and had been on cART longer (11.8 vs 9.2 years and 11 vs7.6 years) (Table 1).

**Table 1.** Baseline Characteristics

	Discovery Cohort (n = 994)		*P* Value	Validation Cohort (n = 200)		*P* Value
	Plaque: 502 (51%)	No Plaque: 492 (50%)		Plaque: 82 (41%)	No Plaque: 112 (49%)	
Age, median (IQR), y	55 (12)	45.5 (17)	<.001	56.5 (12)	48.5 (15)	<.001
Sex, male, n (%)	424 (84)	400 (81)	NS	69 (84)	92 (82)	NS
Race or ethnicity, n (%)						
White	370 (74)	318 (65)	.007	71 (87)	96 (86)	NS
Black	48 (10)	75 (15)		7 (9)	9 (8)	
Asian	32 (6)	26 (5)		3 (4)	2 (2)	
Hispanic	17 (3)	21 (4)		0 (0)	2 (2)	
Mixed	35 (7)	52 (11)		1 (1)	3 (3)	
Body mass index, median (IQR), kg/m^2^	24.8 (5.2)	24.6 (5.4)	NS	25.2 (5.6)	25.7 (6.6)	NS
Smoking status, n (%)						
Current smoking	140 (28)	154 (31)	.014	22 (26.8)	30 (26.8)	NS
Previous smoking	167 (33)	123 (25)		33 (40)	28 (25)	
Never smoking	161 (32)	164 (33)		20 (24)	39 (35)	
Unknown	34 (7)	51 (10)		7 (9)	15 (13)	
Hypertension, n (%)	131 (26)	64 (13)	<.001	24 (29)	23 (21)	NS
Hypercholesterolemia, n (%)	105 (21)	78 (16)	.048	27 (33)	11 (10)	<.001
Metabolic syndrome, n (%)	121 (24)	82 (17)	.005	26 (32)	27 (24)	NS
Lipid-lowering therapy, n (%)	81 (16)	40 (8)	<.001	26 (32)	9 (8)	<.001
CD4 nadir, median (IQR) cells/mm^3^	230 (226)	290 (276)	<.001	225 (263)	275 (252)	NS
Most recent CD4+ T cell count, median (IQR), cells/mm^3^	690 (334)	720 (380)	NS	725 (305)	600 (360)	.003
HIV duration, median (IQR), y	14.6 (12.1)	11.5 (10.9)	<.001	13.7 (11.6)	10.4 (11.5)	.02
cART duration, median (IQR), y	11.8 (10.7)	9.2 (8.7)	<.001	11 (11.3)	7.6 (7.6)	.009

Baseline characteristics of participants with and without carotid plaques in discovery and validation cohort.

Comparison between participants with and without carotid plaques were analyzed using paired Wilcoxon test or *χ*^2^ or (Fisher's exact) when applicable. Statistical significance is *P* < .05.

cART, combined antiretroviral therapy; NS, not significant; IQR, interquartile range.

In the discovery cohort, those with plaques were more likely to be of European ancestry (74% vs 65%), diagnosed with hypertension (26% vs 13%) and metabolic syndrome (24% vs 17%), and had a lower CD4 nadir count (230 vs 290 cells/mm^3^). These differences were not significant in the validation cohort. No differences in body mass index or current smoking status were observed between groups in either cohort.

### Immunophenotyping

**Figure 1.**
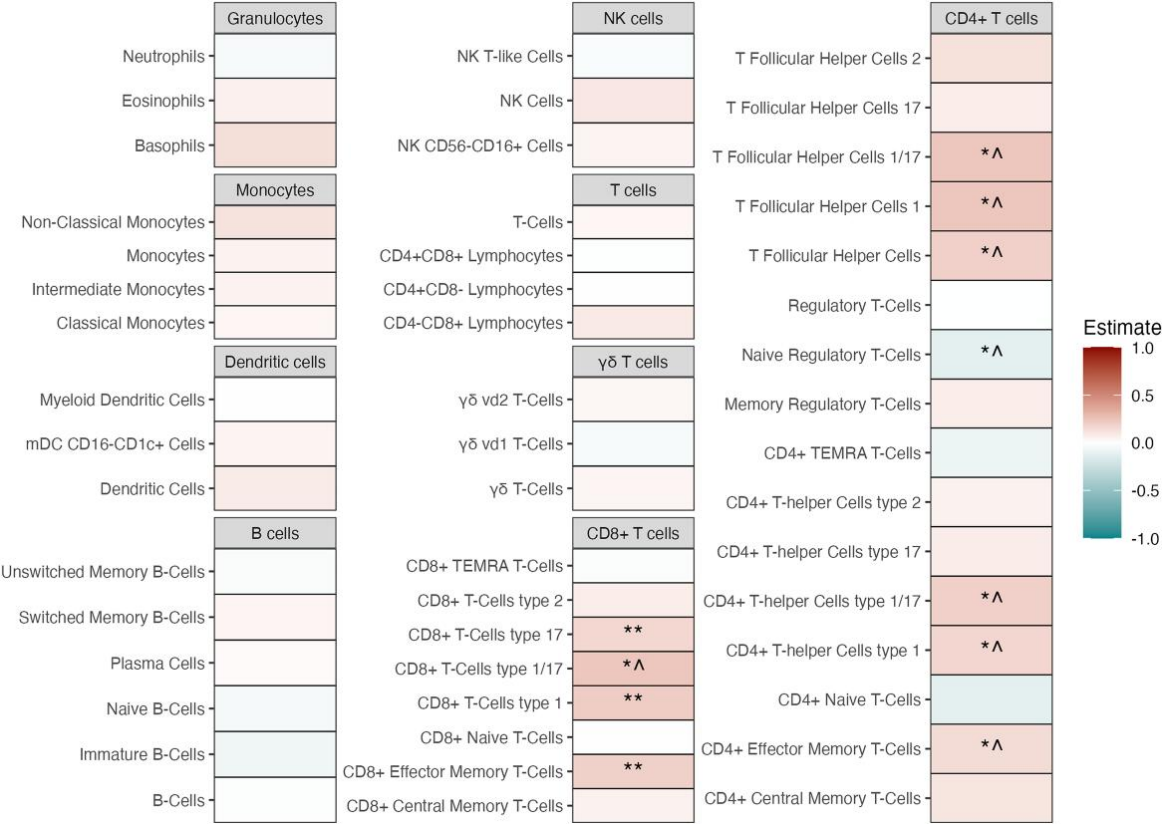
Flow cytometry circulating main cell populations in participants with and without carotid plaques. Comparison of absolute count of major innate and adaptive immune cell population between participants with and without carotid plaques, using estimates from the discovery cohort. Differences were assessed using a linear model adjusted for age, sex, seasonality, current smoking status, use of lipid-lowering drugs, hypertension, metabolic syndrome, and black ethnicity as covariates. A positive Estimate indicates higher levels of the cell population in participants with carotid plaques; negative Estimate indicates lower levels. Symbol definitions: **Significant in both the discovery cohort (FDR < 0.05) and the validation cohort (*P* < .05); *^Significance in the discovery cohort (FDR < 0.05) with the same direction of effect in the validation cohort but without significance (*P* > .05); *#Significance in the discovery cohort (FDR < 0.05) but with an opposite direction of effect in the validation cohort and without significance (*P* > .05). NK cells, natural killer cells; TEMRA, T effector memory cells re-expressing CD45RA; mDC, myeloid dendritic cells.

A total of 335 immune cell subsets were analyzed by flow cytometry (Supplementary Figure 1). Using a linear model corrected for age, sex, seasonality, black ethnicity and cardiovascular risk factors, we found significantly higher absolute counts of CD8^+^ T cell subsets—CD8^+^ T cells 1 (CD8_TC1_), CD8^+^ T cells 17 (CD8_TC17_) and CD8^+^ effector memory T cells (CD8_TEM_) in participants with plaques across both cohorts. In the discovery cohort, additional increases were found in CD8^+^ T cells 1/17 (CD8_TC1/17_) and CD4^+^ T cell subsets, including CD4^+^ T-follicular helper cells 1 (CD4_Tfh1_), T follicular helper cells 1/17 (CD4_Tfh1/17_), CD4^+^ T-helper cells 1 (CD4_Th1_), CD4^+^ T helper cells 1/17 (CD4_Th1/17_), and CD4^+^ effector memory T cells (CD4_TEM_). These trends were present in the validation cohort but not statistically significant (Figure 1, Supplementary Table 4).

**Figure 2.**
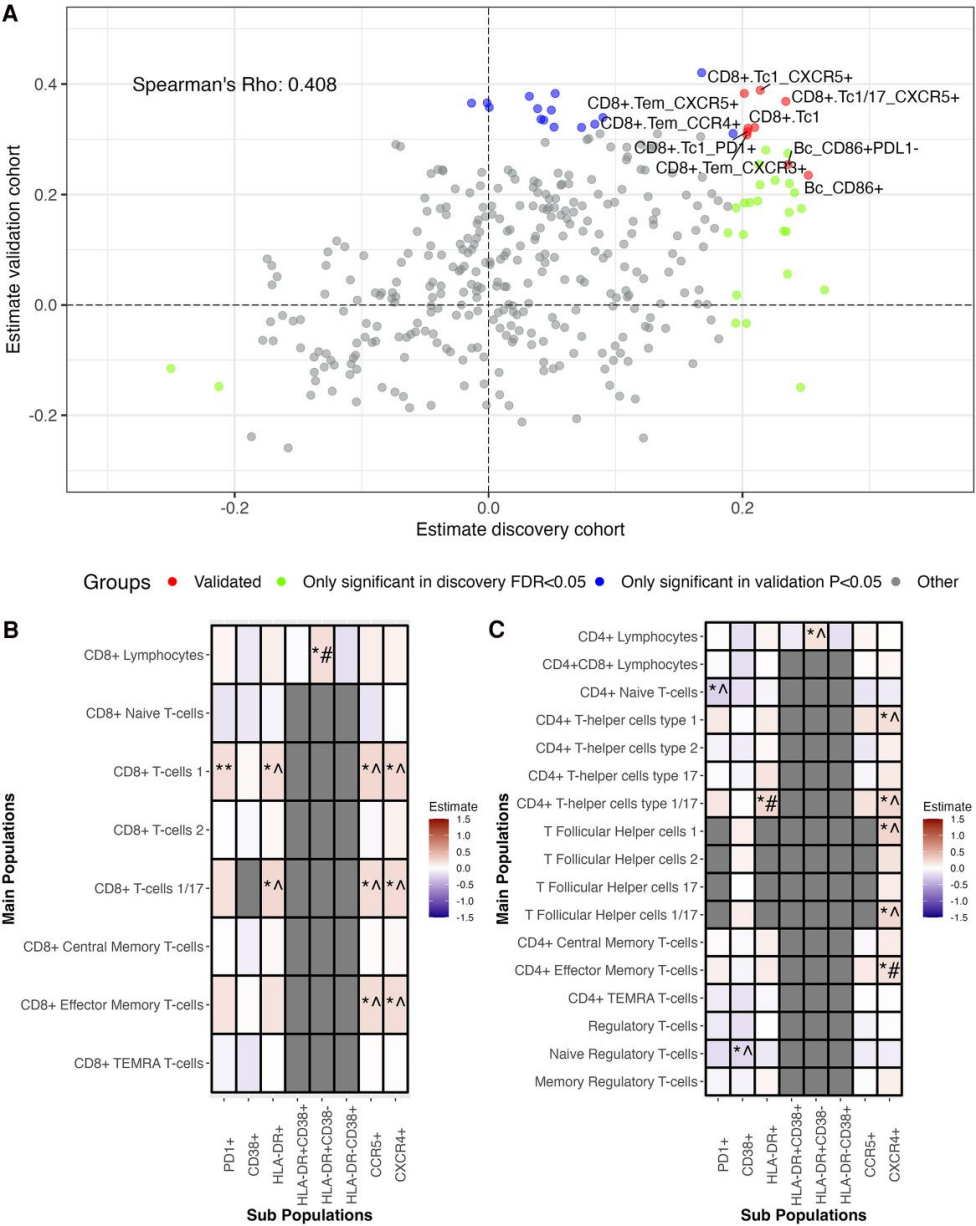
Absolute immune cell counts associated with the presence of carotid plaques. *A*, Correlation of carotid plaque–associated absolute cell subset counts between the discovery and validation cohort, using Spearman's rho. Red dots represent cell populations significantly associated with carotid plaques in both the discovery cohort (FDR < 0.05) and validation cohort (*P* < .05). Green dots represent populations associated with plaques in the discovery cohort (FDR < 0.05) but not in the validation cohort (*P* > .05). Blue dots represent populations significant in the validation cohort (*P* < .05) but not in the discovery cohort (FDR > 0.05). Gray dots represent populations not significant in either cohort (discovery FDR > 0.05 and validation *P* > .05). *B*, CD8^+^ T cell main populations (*Y*-axis) and their defining markers (*X*-axis) associated with the presence of carotid plaques, shown with discovery cohort estimates. *C*, CD4^+^ T cell main populations (*Y*-axis) and their defining markers (*X*-axis) associated with the presence of carotid plaques, shown with discovery cohort estimates. All analyses were adjusted for age, sex, seasonality, current smoking status, use of lipid-lowering drugs, hypertension, metabolic syndrome, and black ethnicity. Symbol definitions: **Significant in both the discovery cohort (FDR < 0.05) and validation cohort (*P* < .05); *^Significant in the discovery cohort (FDR < 0.05) with the same direction of effect in the validation cohort but without significance (*P* > .05); *#Significant in the discovery cohort (FDR < 0.05) but with opposite directionality in the validation cohort and without significance (*P* > .05). Bc_CD86+, CD45^+^, CD3^−^ innate like cell population characterized by CD19^+^, HLA-DR^+^, and CD68^+^ expression, with variable CD36 expression.

Next, we assessed all absolute main cell populations including their receptor expression in the discovery and validation cohort (Figure 2, Supplementary Figure 3, Supplementary Table 4). Overall, a Spearman's rho of 0.408 for estimate concordance between both cohorts was found. Participants with plaques had more CD8_TC1_ cells expressing the exhaustion and cell senescence marker, the programmed cell death protein 1 (PD1). CD8_TC1_, CD8_TC1/17_, and CD8_TEM_ subsets expressing the chemokine receptor CXCR5 were also elevated, as were CD8_TEM_ expressing CCR4 and CXCR3. We identified an atypical CD45^+^ CD3^−^ innate-like cell population characterized by CD19^+^, HLA-DR^−^ and CD68^+^ expression, with variable CD36 expression, which was found at higher levels in participants with carotid plaques in both cohorts (Figure 2*A*, Supplementary Figure 3).

In the discovery cohort, we additionally observed increased numbers of CD8_TC1_ and CD8_TC1/17_ subsets expressing HLA-DR, CCR5, and CXCR4, with similar trends in the validation cohort (Figure 2*B*).

### Transcriptomic Results in Deconvoluted CD8^±^ T Cells

**Figure 3.**
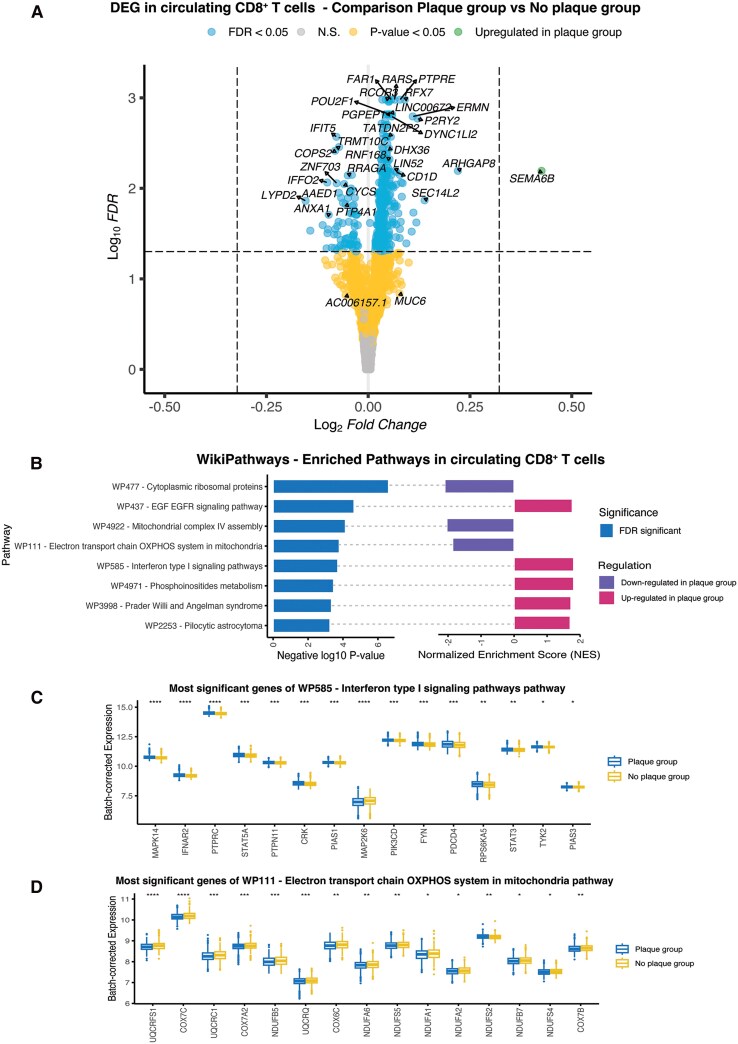
Differential expression of bulk RNA sequencing, deconvoluted for CD8^+^ T cells. *A*, Volcano plot of differentially expressed genes in deconvoluted CD8^+^ T cells from participants with carotid plaques compared with participants without plaques. A linear model was adjusted for age, sex, seasonality, plate number, and ethnicity. The *y*-axis shows FDR-corrected *P* values; the *x*-axis shows the log_2_-fold change. The horizontal dashed line indicates an FDR-corrected *P* <.05. Genes with the lowest FDR-corrected *P* value or largest log_2_-fold change are highlighted. *B*, Enriched pathways identified using Wikipathways. All pathways shown have an FDR-corrected *P* value <.05. Positive NESs indicate upregulated pathways, negative NESs indicate downregulated pathways. *C*, Box plot of batch-corrected expression of the most significant genes in the interferon type 1 signaling pathway from Wikipathways, comparing participants with and without carotid plaques ****P <.0001, ***P <.001, **P <.01, *P <.05. *D*, Box plots of batch-corrected expression of the most significant genes in electron transport chain/OXPHOS pathway from Wikipathways, comparing expression in participants with and without carotid plaques. Significance levels as in (*C*).

CD8^+^ T cell transcriptomes were inferred by deconvolution of PBMCs bulk RNAseq data. Comparing those with and without carotid plaques, 428 genes were significantly differentially expressed in the discovery cohort (FDR < 0.05), including 355 upregulated and 73 downregulated in participants with plaques (Figure 3*A*, Supplementary Table 5).

In the validation cohort, 13 of these genes remained significantly different, 12 of which showed the same direction of change (Supplementary Table 6). Overall, 72% (308/428) of the significant genes from the discovery cohort had consistent expression patterns in the validation cohort (Figure 3*A*, Supplementary Table 5).

### Gene Set Enrichment Analysis

To explore functional pathways, GSEA was conducted using genes from the discovery cohort (Supplementary Tables 7–14). Eight pathways from WikiPathways were significantly enriched (FDR < 0.05), including 5 upregulated and 3 downregulated in plaque carriers (Figure 3*B*, Supplementary Table 7). Among upregulated pathways were Interferon type 1 signaling pathway (normalized enrichment score [NES] = 1.81, *P* = .0002, FDR = 0.028) and epidermal growth factor receptor (EGFR) signaling pathway (NES = 1.76, *P* = 2.31 × 10^−5^, FDR = 0.008). Downregulated pathways included the mitochondrial electron transport chain of oxidative phosphorylation (NES = −1.86, *P* = .0002, FDR = 0.028) and complex IV assembly (NES = −2.04, *P* = 7.20 × 10^−5^, FDR = 0.017) (Figure 3*C*, Supplementary Table 7).

## DISCUSSION

In this study, we examined peripheral immune cell profiles in PLHIV, comparing those with and without carotid plaques, excluding those with prior ASCVD events. We observed higher absolute counts of various CD8^+^ T cell subsets in participants with plaques, particularly CD8^+^ cytotoxic T cells with an interferon secretion profile and CD8^+^ effector memory cells. Among these cells, we found higher counts of CD8^+^ T cells 1, expressing PD1, a marker associated with cell exhaustion and senescence [25, 26]. Transcriptomic analysis of CD8^+^ T cells revealed upregulation of type 1 interferon and EGF/EGFR signaling pathway, and downregulation of mitochondrial functions—features linked to T cell senescence and ageing [27–29].

Adaptive immunity significantly contributes to atherosclerosis [30]. Human carotid plaques show infiltration by both CD4^+^ and CD8^+^ T cells [31], with CD8^+^ T cells more abundant [32]. In mouse models of atherosclerosis, hypercholesterolemia activates CD8^+^ T cells, which then produce cytokines and contribute to plaque formation via cytotoxic mechanisms [33]. Depleting CD8^+^ T cells in atherosclerosis-prone apolipoprotein E-deficient (*Apoe^−/−^)* mice reduces plaque development and inflammation, whereas reintroduction of CD8^+^ T cells reverses the effect [34]. These cells promote macrophage apoptosis and necrotic core formation through granzyme B and perforin [34]. CD8^+^ T cells also enhance monopoiesis, increasing plaque macrophage content without necessarily increasing monocyte recruitment to plaques [35]. However, their role in advanced lesions is more complex, with a study suggesting they may limit lesion progression by restraining CD4^+^ T cell and macrophage accumulation [36].

Several studies with relatively small sample sizes have reported associations between circulating immune cell subsets and subclinical carotid artery disease in PLHIV. In virally suppressed HIV-infected women (n = 29), higher proportions of activated CD4^+^ and CD8^+^ T cells (CD38^+^HLA-DR^+^) and immunosenescent CD8^+^ T cells (CD28^−^CD57^+^) were associated with carotid artery plaques [37]. Although we used different markers to characterize immunosenescence, we did observe an association between PD1 expression on CD8^+^ cytotoxic T cells and plaque presence. As PD1 is also expressed on senescent cells, this finding supports a potential link between immune ageing and atherosclerosis.

Another study reported correlations between activated CD8^+^ T cells (CD38^+^HLA-DR^+^) and common carotid artery intima-media thickness (IMT) in virally suppressed PLHIV (n = 60) [38]. In contrast, we were unable to replicate associations with activated CD4^+^ and CD8^+^ T cells. Several factors may explain this discrepancy. First, our analysis included 355 immune cell subsets, requiring correction for multiple testing, which increases statistical stringency and may attenuate weaker associations. Second, our cohort was substantially larger and more heterogeneous, with an overrepresentation of men, potentially diluting effects observed in smaller, more homogeneous populations. Finally, methodological differences in sample processing and phenotyping panels may also contribute. Similarly, associations reported between activated memory CD8^+^ T cells (CD38^+^CD45RO^+^), apoptotic CD4^+^ and CD8^+^ T cells (CD95^+^) and increased IMT (≥1 mm) in PLHIV (n = 163) could not be replicated due to differences in phenotyping strategies [39]. The Veterans Aging Cohort Study identified associations between several CD4+ T cell subsets and incident cardiovascular disease in PLHIV (n = 1269) [40]. We did not observe similar associations with subclinical disease, which may reflect differences in population characteristics, including ethnicity, and distinct BPs underlying plaque presence versus clinical cardiovascular events.

Notably, in both cohorts, we identified higher levels of an atypical CD45^+^CD3^−^ cell population expressing CD19, HLA-DR and CD58, with variable CD36 expression. This mixed antigen-presenting and phagocytic phenotype suggests a previously undercharacterized immune population associated with carotid plaque presence.

An important observation in our study was the increased presence of PD-1 expressing CD8^+^ cytotoxic T cells in participants with plaques. PD-1 is an inhibitory receptor that is upregulated during chronic antigen exposure and is associated with T cell exhaustion and senescence. In PLHIV, several factors contribute to persistent immune activation, including the latent HIV reservoir, microbial translocation, co-infections, clonal hematopoiesis, and possibly ART itself [41]. These processes may collectively drive CD8+ T cell senescence. In non-small cell lung cancer, PD-ligand 1 (PD-L1) expression has been linked to activation of the EGFR signaling pathway [29], which we also found to be upregulated in CD8^+^ T cells from participants with plaques, suggesting shared immunometabolic mechanisms.

Another hallmark of T cell senescence is mitochondrial dysfunction, characterized by impaired electron transport chain activity and reduced complex IV assembly [27, 42, 43]. Such mitochondrial abnormalities have been described in PLHIV, despite long-term viral suppression on ART [44], and are associated with premature ageing and age-related comorbidities, including cardiovascular disease [45]. Experimental studies further support a causal role for senescent CD8^+^ T cells in atherosclerosis. Depletion of CD8^+^ T cells reduces atherosclerotic lesion development in aged mice, while transfer of CD8^+^ T cells accelerates disease in CD8-deficient mice [46].

We also observed upregulation of type I interferon signaling in CD8^+^ T cells, a pathway that contributes to cellular senescence [47] and the pro-inflammatory senescence-associated secretory phenotype [28]. Type 1 interferons activate endothelial and immune cells, promote foam cell formation, and facilitate leukocyte recruitment to plaques [48]. Consistent with this, autoimmune diseases with heightened interferon activity, such as systemic lupus erythematosus, are associated with increased cardiovascular risk.

Despite transcriptomic evidence pointing toward a senescent and cytotoxic CD8+ T cell phenotype, we did not find a significant association between terminally differentiated effector memory (TEMRA) CD8^+^ T cells and plaque presence. Specifically, CD45RA-expressing CD8^+^ T cells were not associated with carotid plaques. A likely explanation is that CD45RA alone does not uniquely define senescent CD8^+^ T cells. CD45RA can be re-expressed on several CD8^+^ T cell states, including heterogeneous TEMRA populations that comprise terminally differentiated, exhausted and effector-like cells. As a result, CD45RA expression lacks the specificity required to capture the transcriptionally senescent phenotype, identified in our bulk CD8^+^ T cell transcriptomic analyses, suggesting that senescence-related transcriptional signature may arise from a subset of CD8^+^ T cells not reliably distinguishable using surface markers alone.

The prevalence of carotid plaques varies wildly across studies of PLHIV [49, 50] and was relatively high in our cohort. Direct comparison between studies is challenging due to differences in population characteristics, imaging techniques, and plaque definitions. Importantly, we adhered to international guidelines on plaque reporting [15].

This study has several strengths. It includes a large multicenter cohort from 4 Dutch centers, divided into a discovery and validation cohort, enabling robust confirmation of findings. Comprehensive immunophenotyping enabled identification of specific immune cell subsets associated with plaque presence. Uniform training of ultrasound assessors minimized diagnostic variability. Additionally, deconvoluted RNA sequencing of CD8^+^ T cells provided functional insight into immune pathways associated with subclinical atherosclerosis.

Several limitations should be acknowledged. The cross-sectional design precludes causal inference, making it unclear whether immune dysregulation precedes or follows plaque development. Certain ART regimens, particularly abacavir-containing therapies, have been associated with cardiovascular risk. We did not include ART regimen in our models, as recent treatment changes may not reflect cumulative exposure and could introduce misclassification. Most participants were treated with contemporary ART regimens, predominantly INSTI-based combinations [14]. Carotid ultrasound cannot distinguish stable from vulnerable plaques, limiting insight into plaque instability. The cohort was predominantly male (82%), restricting generalizability to women living with HIV. Differences between discovery and validation cohorts in HIV-related characteristics and plaque prevalence could not be fully explained, despite reevaluation of ultrasound images. Single-cell sequencing was not available, necessitating the use of bulk RNA deconvolution, which provides inferred rather than directly measured cell proportions. We must acknowledge that the magnitude of difference in gene expression between plaque and non-plaque is small, driven by the large sample size of genes. Finally, although pandemic-related factors were considered, unmeasured effects of the COVID-19 period may have influenced immune profiles.

In conclusion, we identified that PLHIV with carotid plaques exhibit increased circulating CD8^+^ T cell subsets, particularly those with a type 1 interferon secreting signature and markers of exhaustion and senescence. Transcriptomic analysis supported a role for mitochondrial dysfunction, interferon signaling, and EGFR pathway activation in these cells. These findings underscore the contribution of immune senescence to atherosclerosis in PLHIV and may offer targets for future therapeutic strategies to reduce cardiovascular risk in this population.

## Supplementary Material

jiag018_Supplementary_Data

## Supplementary Data

Supplementary materials are available at *The Journal of Infectious Diseases* online (http://jid.oxfordjournals.org/). Supplementary materials consist of data provided by the author that are published to benefit the reader. The posted materials are not copyedited. The contents of all supplementary data are the sole responsibility of the authors. Questions or messages regarding errors should be addressed to the author.

## Notes

***Acknowledgments.*** We thank all participants and the research teams of 2000HIV studies within the Human Functional Genomics Project for their collaboration and support. We thank the Stichting HIV Monitoring for providing us with relevant clinical data from the AIDS Therapy Evaluation in the Netherlands (ATHENA) cohort. The ATHENA database is managed by Stichting HIV Monitoring and supported by a grant from the Dutch Ministry of Health, Welfare and Sport through Center for infectious disease control of the National Institute for Public Health and the Environment. The data underlying this article will be shared on reasonable request to the corresponding author.

***Author Contributions.*** M. J. T. B. and V. R. V. performed the analysis of the data and wrote the manuscript. M. J. T. B., W. A. J. W. V., A. L. G., and L. E. v. E. collected the data. A. N. performed the flow cytometry analysis. G. W. performed quality control of the ultrasound data. M. A. H. B., A. J. A. M. v. d. V., J. H. W. R., and N. P. R. supervised the work. A. J. A. M. v. d. V. and M. G. N. designed the project. All authors contributed to manuscript revision and read and approved the submitted version.

***Financial support.*** This work was supported by ViiV Healthcare (A18-1052). ViiV Healthcare grand awarded to A. J. A. M. v. d. V. Although there is close collaboration, ViiV Healthcare did not have any role in data quality control, statistical analysis, and final interpretation of the data.
