# Supplementary material for: Functional and Transcriptional Senescence Profiles of CD8+ T Cells Associate With the Presence of Carotid Plaques in People Living With HIV
Source: J Infect Dis. 2026 Jan 7;233(5):e1154–64. doi: 10.1093/infdis/jiag018 (PMC13175616; doi:10.1093/infdis/jiag018)
Supplement: jiag018_Supplementary_Data [file jiag018_supplementary_data.zip › Supplementary figures.pdf]

Supplementarty Figure 1: Overview of circulation immune cell populations

Immunophenotype of circulating immune cell populations evaluated with flow cytometry panel 1 - Innate immune cells

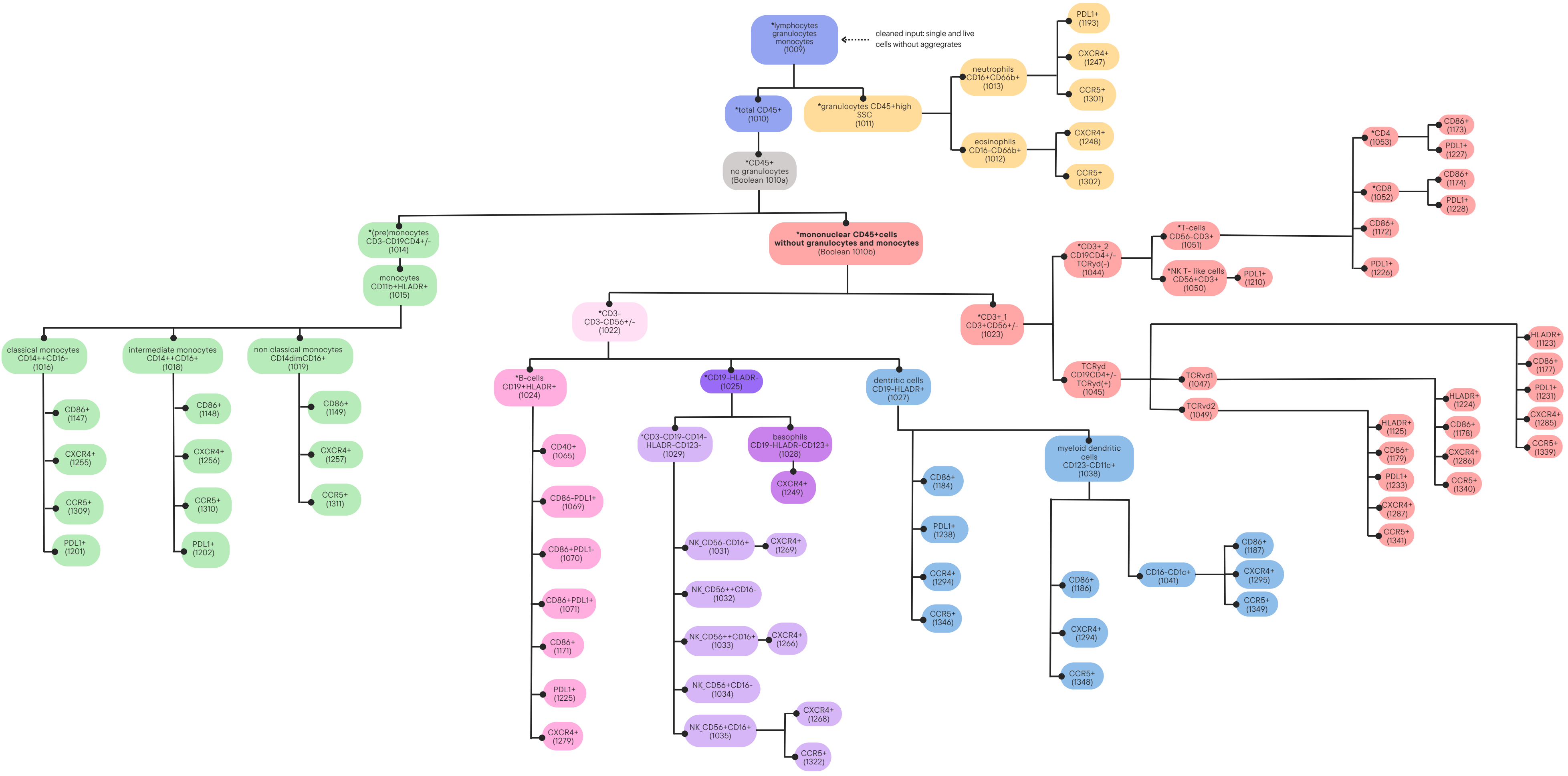

(*) population not present in the filtered_3 data set

Immunophenotype of circulating immune cell populations evaluated with flow cytometry panel 2 - T lymfocytes

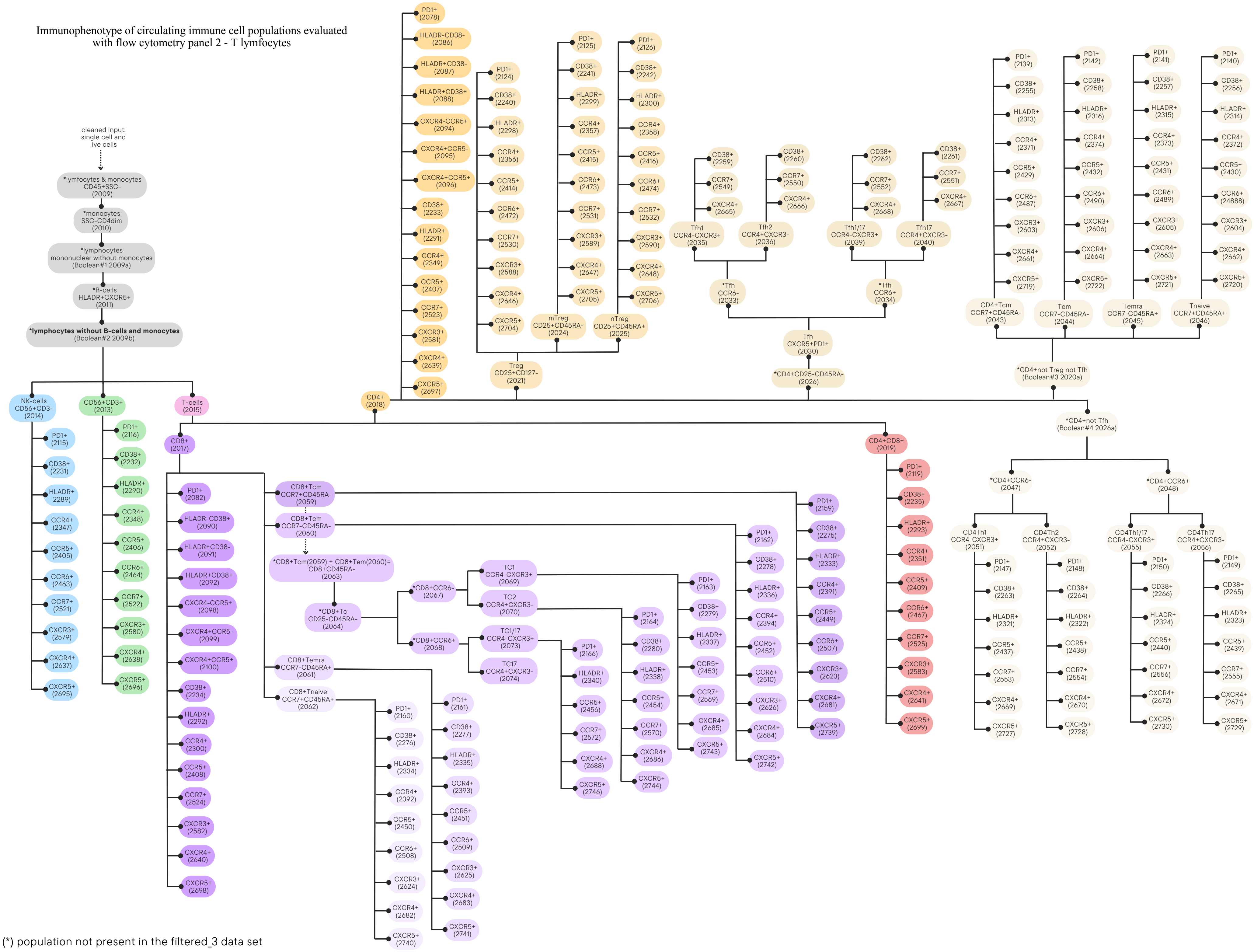

(*) population not present in the filtered_3 data set

Immunophenotype of circulating immune cell populations evaluated with flow cytometry panel 3 - B-lymfocytes

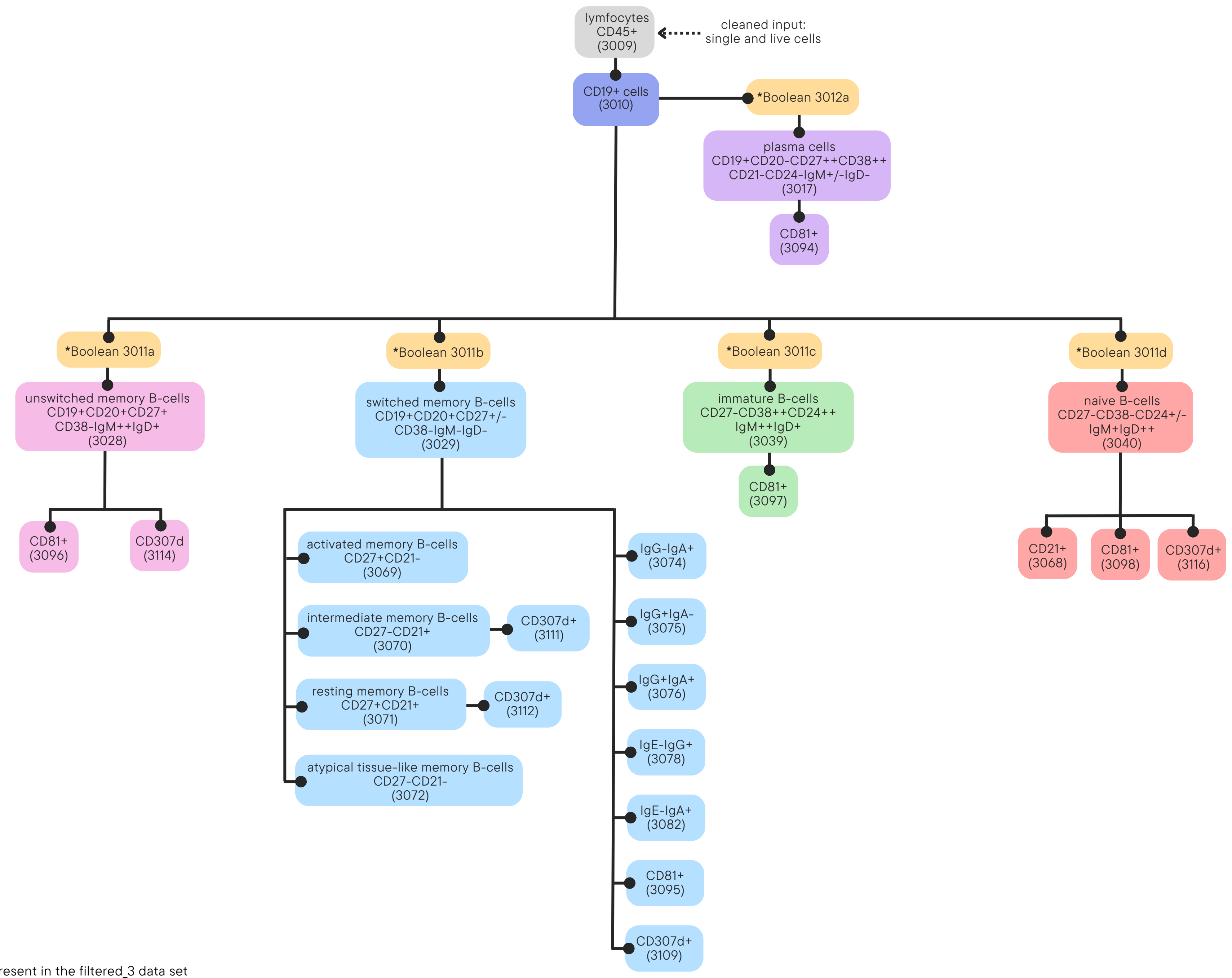

(*) population not present in the filtered_3 data set

Figure S2: Principal component analysis on potential confounders of flow cytometry absolute counts data.

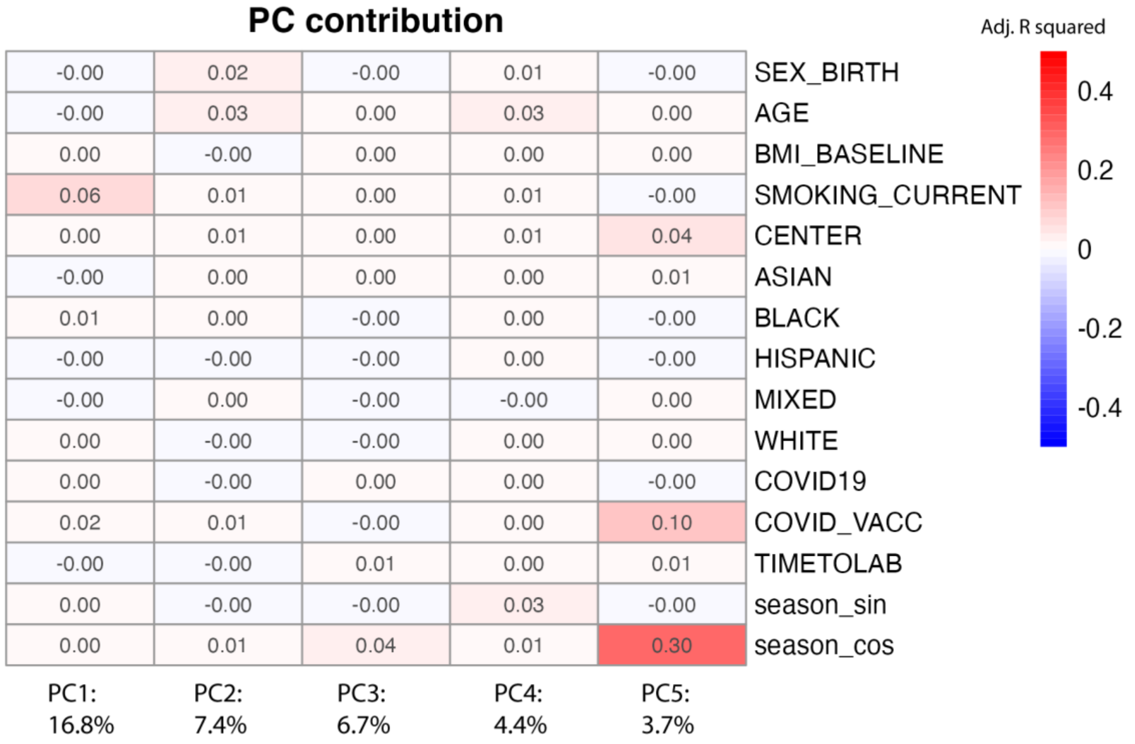

Principal component analysis on potential confounders of flow cytometry data. The first 5 principal components (PCs) are shown. The results are presented as adjusted R^2 values with variables having high adjusted R^2 values considered as potential confounders. Seasonality (season_cos) appeared to influence flow cytometry results and were therefore corrected for in the analysis.

Figure S3: Violin plots of absolute counts of different immune cell populations comparing participants with and without carotid plaques

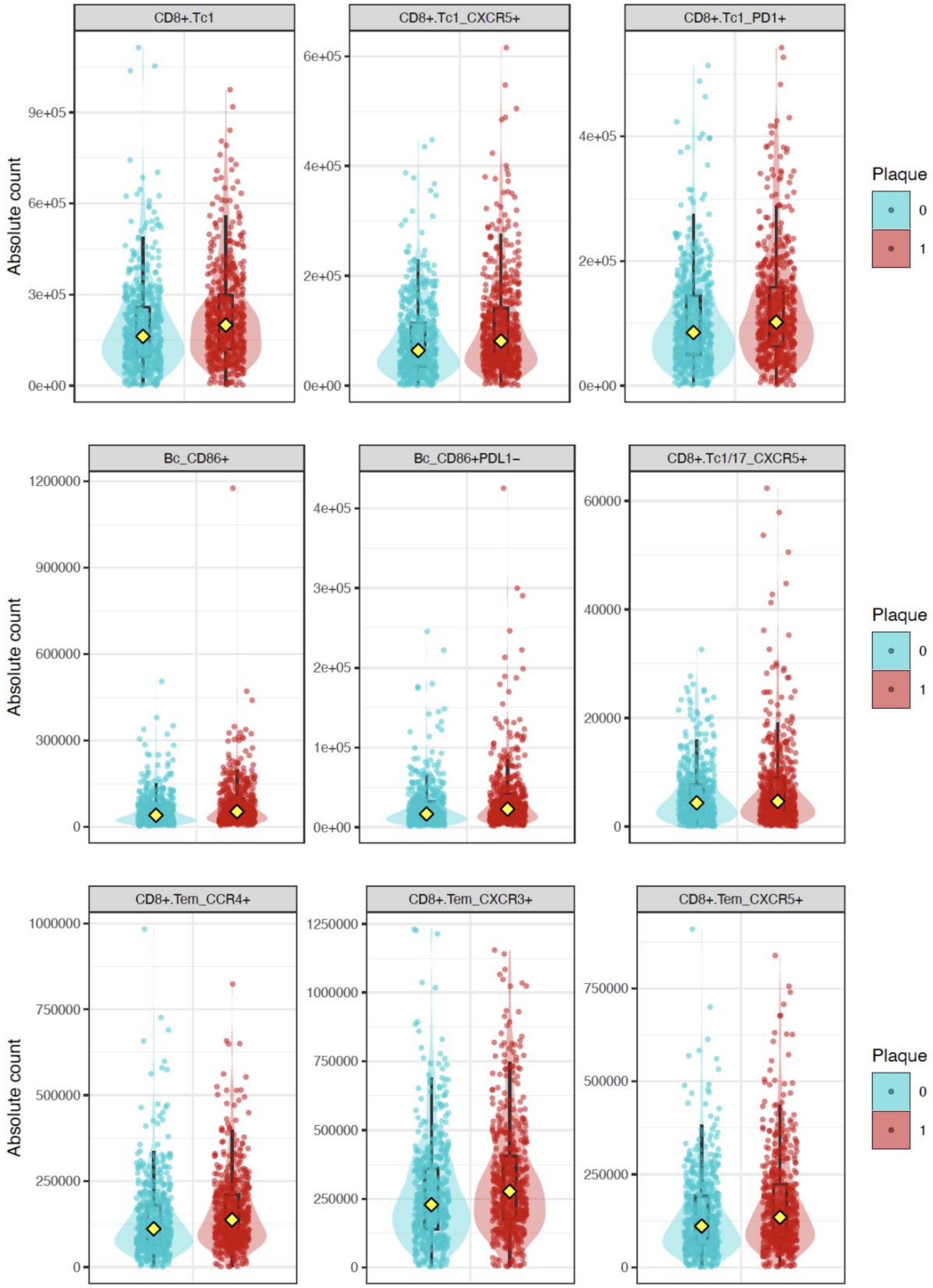

Violin plots illustrating the distribution of absolute counts of $CD8^+Tc_1$, $CD8^+Tc_1CXCR5^+$, $CD8^+Tc_1PD1^+$, $CD8^+Tc_{1/17}$ $CXCR5^+$, Bc $CD86^+$, Bc $CD86^+$ $PDL1^-$, $CD8^+$Tem $CCR4^+$, $CD8^+$Tem $CXCR3^+$ and $CD8^+$Tem $CXCR5^+$ comparing those with (red) and without (turquoise) carotid plaques. Yellow represents the median value.

Figure S4: Absolute counts of CD8⁺ and CD4⁺ T cell main populations with receptor markers comparing those with and without carotid plaques.

A

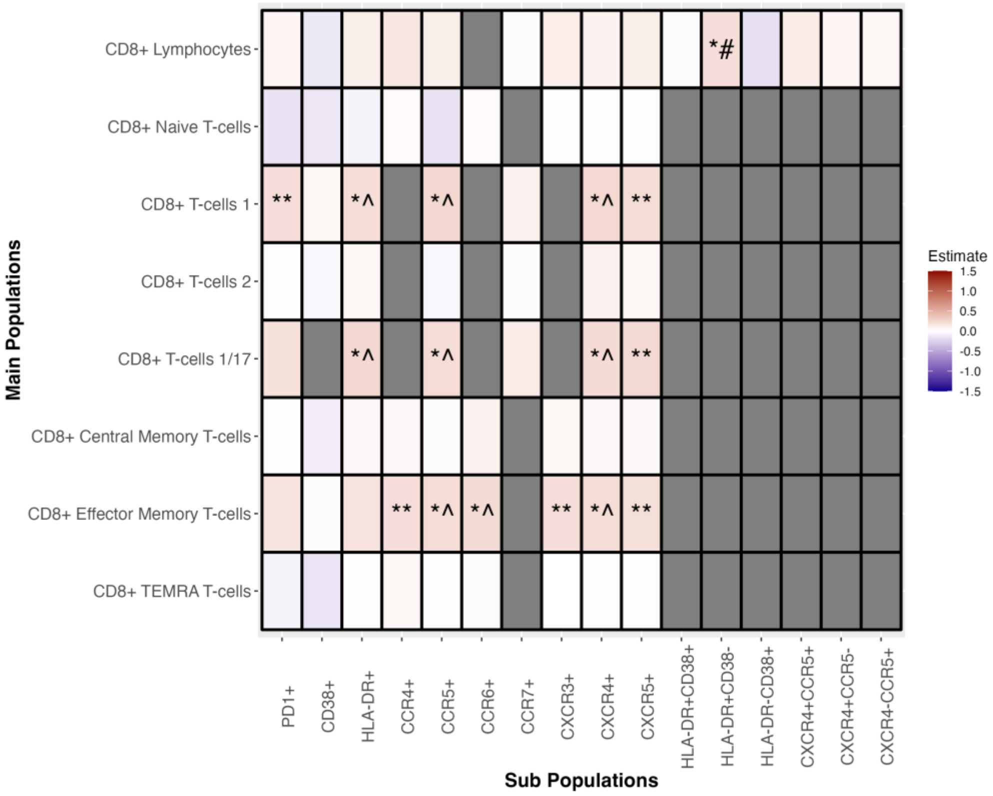

B

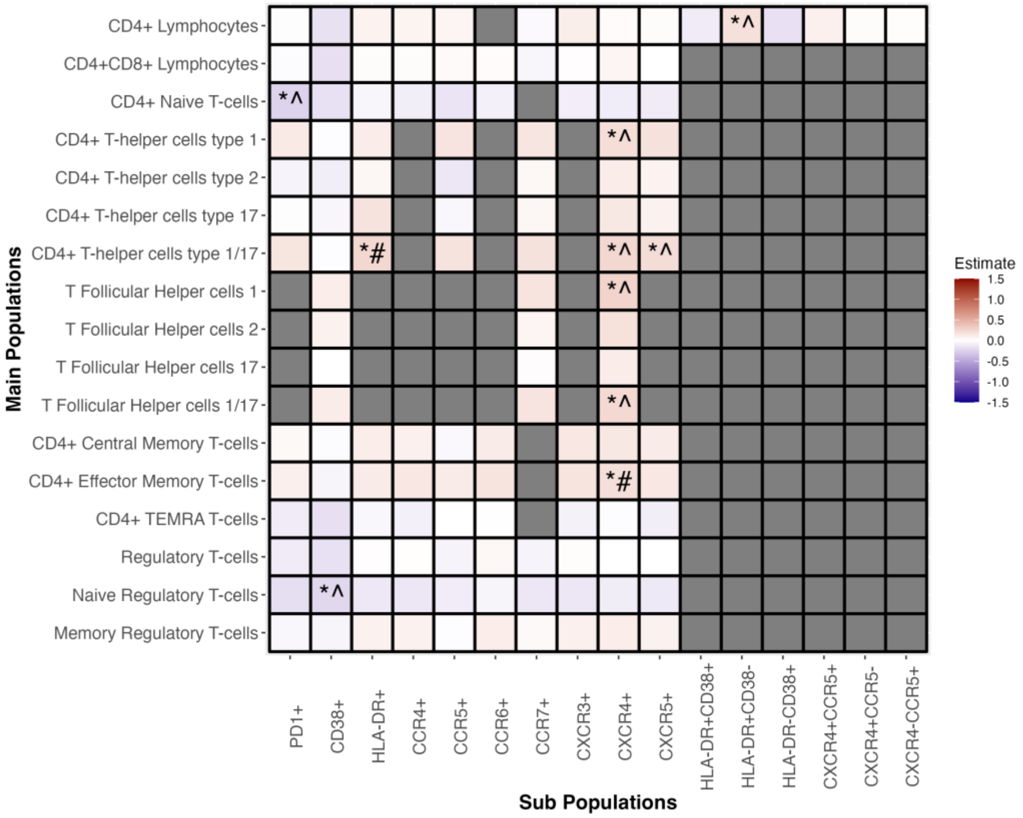

Results from the discovery and validation are shown, using the estimate from the discovery cohort. The y-axis displays the main CD8⁺ T-cell population **(A)** and CD4⁺ T cell

population **(B)**, and the x-axis shows the receptor markers defining each subpopulation. Associations were tested using linear regression models adjusted for age, sex, seasonality, current smoking status, medical history of hypertension, use of lipid lowering drugs, metabolic syndrome and black ethnicity as covariates. Effect estimates (discovery cohort) are shown in red (higher in participants with plaques) and purple (lower in participants with plaques).

** indicates a significant difference between participants with and without carotid plaques in both the discovery and validation cohorts.
*^ indicates significance in the discovery cohort (FDR < 0.05) with the same direction of effect in the validation cohort, but not statically significant (*P* > 0.05)
*# indicates significance in the discovery cohort (FDR < 0.05) but with an opposite direction in the validation cohort without significance (*P* > 0.05).

Figure S5: Violin plots of CD4 count distribution for both cohorts

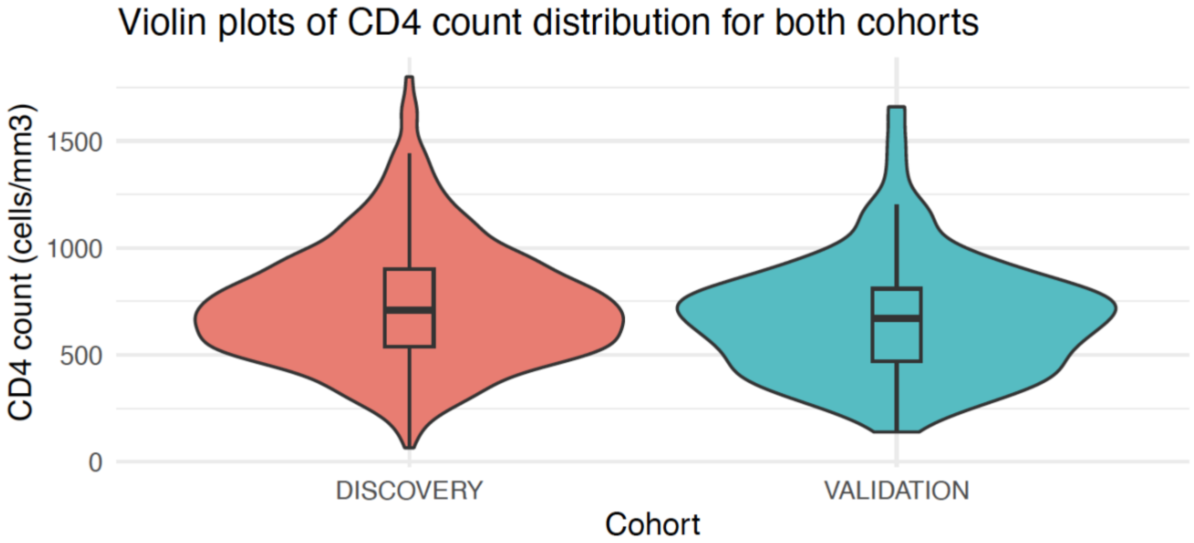

CD4 count of discovery cohort (red) and validation cohort (turquoise)
